# Novel Biomarkers for Improving the Diagnosis of Appendicitis in Pediatric Patients

**DOI:** 10.1002/jcla.70048

**Published:** 2025-05-03

**Authors:** Adir Alper, Yizhak Aronov, Lior Shlomov, Osnat Zmora

**Affiliations:** ^1^ Shamir Medical Center Be'er Ya'acov Israel; ^2^ Department of Pediatric Surgery Shamir Medical Center Be'er Ya'acov Israel

**Keywords:** appendicitis/diagnosis, biomarkers, C‐reactive protein, pediatrics, ROC curve

## Abstract

**Introduction:**

Diagnosing appendicitis in pediatric patients remains a clinical challenge, especially in resource‐limited settings where imaging tools are less accessible. Inflammatory markers, including the Neutrophil‐to‐Lymphocyte Ratio (NLR), Monocyte‐to‐Lymphocyte Ratio (MLR), Neutrophil‐to‐Monocyte Ratio (NMR), Platelet‐to‐Lymphocyte Ratio (PLR), Neutrophil‐to‐Platelet Ratio (NPR), and C‐reactive protein (CRP), offer a promising approach to enhancing diagnostic accuracy. We aimed to evaluate the utility of these inflammatory markers to diagnose appendicitis.

**Methods:**

This retrospective study included 1027 pediatric patients who underwent appendectomy, with appendicitis confirmed histopathologically in 891 cases. Preoperative Inflammatory markers (NLR, MLR, NMR, PLR, NPR, and CRP) were analyzed and optimal cutoff values were determined using Receiver Operating Characteristic (ROC) curves.

**Results:**

Elevated NLR, NMR, PLR, NPR, and CRP were strongly associated with appendicitis, while an inverse relationship was observed with MLR. NLR (≥ 4.42) and NPR (≥ 0.0327) demonstrated high diagnostic accuracy with sensitivity and specificity exceeding 75%. Surprisingly, MLR showed a statistically significant inverse relationship with AA risk. Temperature differences between groups were not statistically significant.

**Conclusion:**

Each suggested novel inflammatory marker has the potential to improve the preoperative diagnosis of appendicitis in pediatric patients. Such a system could minimize reliance on imaging and expedite decision‐making, especially in resource‐constrained settings. Further prospective studies are needed to validate these findings and explore their clinical utility.

## Graphical abstract

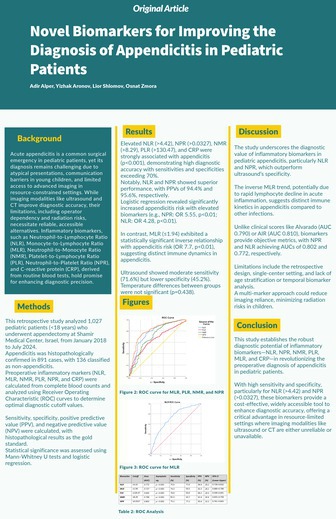

## 1 Introduction

Appendicitis is a common and urgent surgical condition in pediatric patients, with timely diagnosis being crucial to avoid complications such as perforation and peritonitis [1, 2, 3]. Although advances in medical imaging and laboratory diagnostics have improved diagnostic accuracy, pediatric appendicitis remains difficult to evaluate due to atypical symptoms, communication barriers in younger children, and overlapping presentations with other abdominal conditions [4, 5].

Imaging modalities such as ultrasonography (US) and computed tomography (CT) are used to supplement the clinical assessment. While CT offers high diagnostic sensitivity and specificity, its use in children is limited due to radiation risks and the desire to adhere to ALARA (As Low As Reasonably Achievable) principles [6]. Although US avoids radiation and is widely available, its diagnostic yield depends on operator expertise and may be suboptimal in certain clinical situations such as obesity or excessive bowel gas [7]. In light of these limitations, a reliable and easily accessible diagnostic tool is essential to enhance early detection and support informed clinician decision‐making.

Recent studies have highlighted the potential role of inflammatory markers such as the Neutrophil‐to‐Lymphocyte Ratio (NLR), Monocyte‐to‐Lymphocyte Ratio (MLR), and C‐reactive protein (CRP) in diagnosing appendicitis in adults [8, 9]. These markers reflect the body's inflammatory response and can be readily obtained from routine blood tests [10]. NLR has been identified as a significant predictor of appendicitis due to its ability to reflect the balance between neutrophil‐mediated inflammation and lymphocyte‐mediated immune response [11]. Elevated NLR and CRP have been consistently associated with increased likelihood of appendicitis and may also predict complicated cases [9, 12, 13, 14, 15, 16]. Similarly, MLR emerges as an important marker, and assists in differentiating appendicitis from other causes of abdominal pain [17]. Incorporating CRP into a scoring system alongside NLR and MLR may provide a more comprehensive assessment of the patient's inflammatory status [11].

These biomarkers may be combined with clinical decision tools such as the Alvarado score or the Appendicitis Inflammatory Response (AIR) score, which integrate laboratory data and clinical symptoms to guide management [18, 19].

Elevated body temperature is useful in evaluating patients with suspected appendicitis. However, temperature rise is higher in young children under 5 than in older children [20, 21, 22, 23].

Importantly, recent studies suggest that biomarkers may be useful not only in confirming appendicitis but also in differentiating between uncomplicated and complicated appendicitis, potentially aiding in non‐operative management strategies [24, 25]. Inflammatory biomarkers such as the neutrophil‐to‐lymphocyte ratio (NLR), platelet‐to‐lymphocyte ratio (PLR), and monocyte‐to‐lymphocyte ratio (MLR) have gained attention for their utility in diverse clinical settings such as assessing the magnitude of surgical trauma and predicting postoperative complications. Elevated NLR levels, in particular, have been shown to correlate with the invasiveness of surgical procedures and may serve as early indicators of adverse outcomes, including infections and delayed recovery. Moreover, these biomarkers have demonstrated clinical relevance in differentiating periprosthetic joint infections (PJI) from aseptic failures following orthopedic surgeries and in identifying early stages of sepsis, thus enhancing diagnostic accuracy and enabling timely therapeutic interventions [26, 27]. The expanding role of inflammatory biomarkers in oncology, autoimmune disease, and infectious diseases such as sepsis further underscores their diagnostic value [28, 29, 30, 31].

The integration of these inflammatory markers into a scoring system could potentially reduce the dependency on imaging, particularly in settings where access to advanced diagnostic tools is limited. This study aims to develop and validate a novel scoring system that incorporates NLR, NPR, NMR, PLR, MLR, CRP levels, and temperature to improve the diagnostic accuracy of appendicitis in pediatric patients.

## 2 Materials and Methods

### 2.1 Study Design and Population

We conducted a retrospective study of pediatric patients (aged < 18 years) who underwent appendectomy at Shamir Medical Center from January 2018 to July 2024. The preoperative diagnosis of appendicitis was made by pediatric surgeons based on a combination of clinical signs and symptoms such as abdominal pain migrating to the right lower quadrant associated with nausea, anorexia, rebound tenderness, and guarding; lab results, such as elevated CRP and white blood cell counts, and ultrasound findings. The study included children with appendicitis of varying severity, ranging from early‐stage uncomplicated appendicitis to complicated cases with perforation or abscess formation. The study was approved by the Shamir Medical Center Institutional Review Board (IRB), ensuring it met all ethical requirements.

### 2.2 Inclusion and Exclusion Criteria

Included were patients with complete preoperative blood counts and histopathology results. Excluded were pregnant patients, those with chronic inflammatory or infectious diseases (e.g., inflammatory bowel disease), or incomplete data (missing imaging or laboratory values).

### 2.3 Data Collection

Data included age, sex, temperature, preoperative complete blood count (CBC) parameters (neutrophils, lymphocytes, monocytes, and platelets), CRP levels, US findings, and histopathology reports. Appendicitis cases included simple and complicated (e.g., necrosis and perforation) per histopathology as one group. Non‐appendicitis (NA) cases included normal appendices, subsiding inflammation, *Enterobius vermicularis* , and follicular lymphoid hyperplasia.

### 2.4 Biomarker Calculations

Biomarkers were derived from CBC results (Sysmex XN‐1000 analyzer):

$$\begin{array}{c} \mathrm{NLR}=\text{Neutrophils}\;\left(K/\mathrm{\mu L}\right)/\text{Lymphocytes}\;\left(K/\mathrm{\mu L}\right)\\ \mathrm{MLR}=\text{Monocytes}\;\left(K/\mathrm{\mu L}\right)/\text{Lymphocytes}\;\left(K/\mathrm{\mu L}\right)\\ \begin{array}{c} \mathrm{NMR}=\text{Neutrophils}\;\left(K/\mathrm{\mu L}\right)/\text{Monocytes}\;\left(K/\mathrm{\mu L}\right)\\ \begin{array}{c} \mathrm{PLR}=\text{Platelets}\;\left(K/\mathrm{\mu L}\right)/\text{Lymphocytes}\;\left(K/\mathrm{\mu L}\right)\\ \mathrm{NPR}=\text{Neutrophils}\;\left(K/\mathrm{\mu L}\right)/\text{Platelets}\;\left(K/\mathrm{\mu L}\right) \end{array} \end{array} \end{array}$$

### 2.5 Statistical Analysis

Statistical analyses were conducted to examine the relationship between blood biomarkers and pathological findings. Prior to selecting the appropriate tests, data normality was assessed using the Kolmogorov–Smirnov test and Q‐Q plot. Depending on distribution and group size, comparisons between groups were performed using either parametric analysis (T‐test) or non‐parametric analysis (Mann–Whitney *U* test). To evaluate the diagnostic accuracy of various biomarkers, Receiver Operating Characteristic (ROC) curves were generated, and the Area Under the Curve (AUC) was calculated. The AUC analysis specifically compared two groups: appendicitis and Non Appendicitis (NA). Sensitivity, specificity, positive predictive value (PPV), and negative predictive value (NPV) with 95% confidence intervals (CI) were determined for each biomarker and ultrasound finding, with histopathological results serving as the gold standard. All statistical analyses were performed using IBM SPSS Statistics v27.0 (IBM Corp., Armonk, NY, USA).

## 3 Results

**FIGURE 1:**
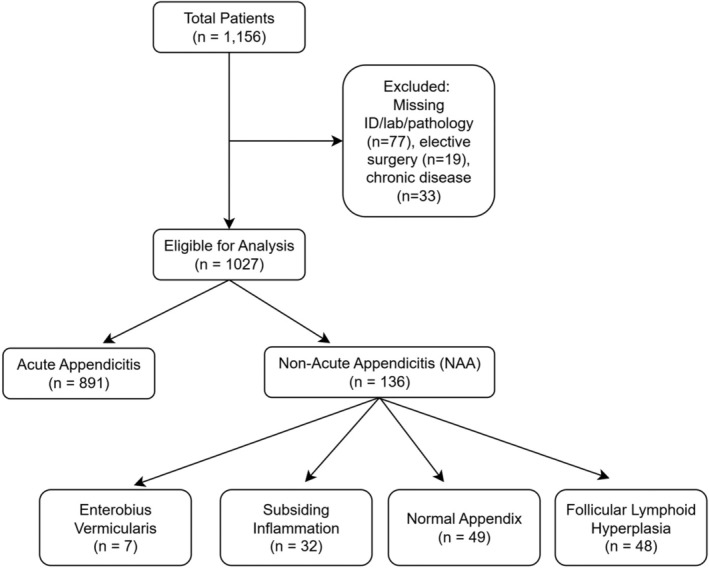
Flow Diagram of Study Population.

Out of 1156 patients who underwent appendectomy, 129 were excluded due to missing data (*n* = 77), elective surgery (*n* = 19), or chronic disease (*n* = 33), leaving 1027 patients eligible for analysis. Among these, 891 patients (86.8%) had histopathologically confirmed appendicitis. The remaining 136 patients (13.2%) were classified as non‐appendicitis cases, which included subsiding inflammation (*n* = 32), normal appendix (*n* = 49), follicular lymphoid hyperplasia (*n* = 48), and *Enterobius vermicularis* infection (*n* = 7) (Figure 1).

Among 891 patients with a histopathological diagnosis of appendicitis, 638 patients (71.6%) had positive ultrasound (US) findings consistent with appendicitis, while 253 patients (28.4%) had negative US findings. The diagnostic performance of ultrasound demonstrated a sensitivity of 71.6% and a specificity of 45.2%.

**FIGURE 2:**
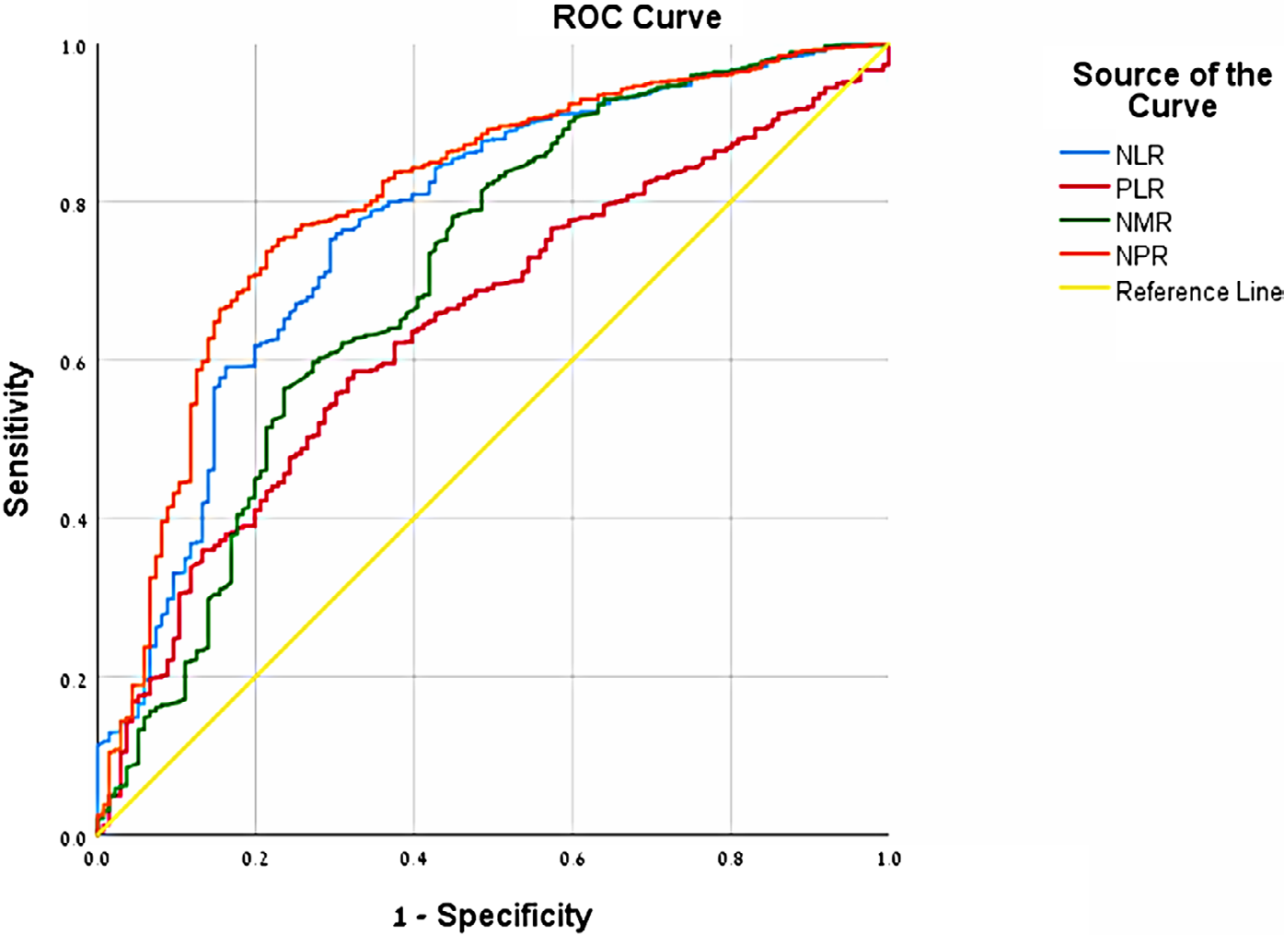
ROC curve for *MLR*, *PLR*, *NMR*, and *NPR*—simulates in graph the relationship between true‐positive rate (sensitivity) and false‐positive rate (1‐specifity).

**FIGURE 3:**
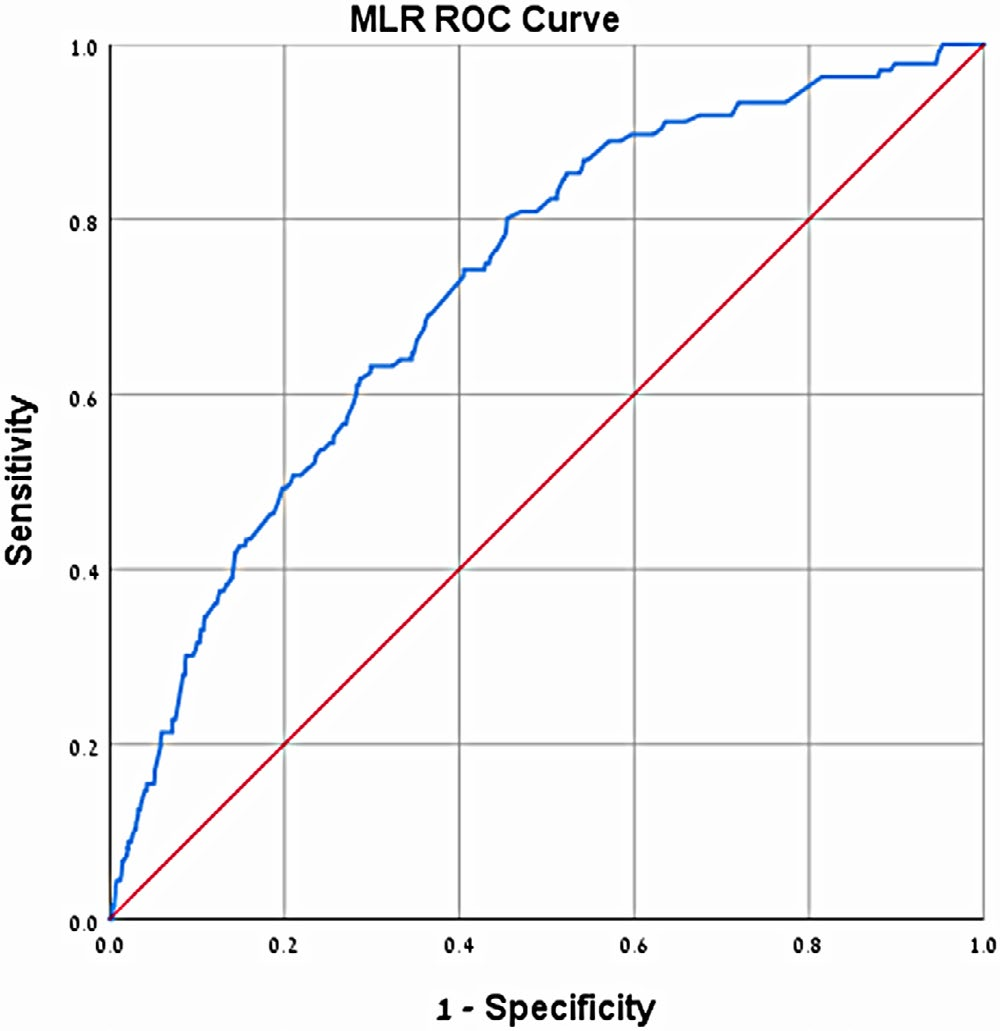
ROC curve for MLR—simulates in graph the relationship between true‐positive rate (sensitivity) and false‐positive rate (1‐specifity) for MLR.

Absolute neutrophil count (ANC), absolute platelet count (APC), absolute monocyte count (AMC), CRP, NLR, PLR, NMR, NPR were higher in the appendicitis group, whereas absolute lymphocyte count (ALC) and MLR were lower in the appendicitis group (Table 1). The only tested variable that did not show statistically significant differences between the groups was temperature (Table 1). We suggest five cut‐off values: for NLR of 4.419 (sensitivity 75%, specificity 71%, PPV 94.4%, NPV 30.2%), for NPR of 0.0327 (sensitivity 75.2%, specificity 77.2%, PPV 95.6%, NPV 32.2%), for NMR of 8.29 (sensitivity 82.3%, specificity 50.7%, PPV 91.6%, NPV 30.4%), for PLR of 130.47 (sensitivity 70%, specificity 50%, PPV 90.2%, NPV 20.3%), and for MLR of 1.94 (sensitivity 74.3%, specificity 59.5%, PPV 92.3%, NPV 26.1%). These values represent the most appropriate combination of high sensitivity, specificity, positive predictive value (PPV), and negative predictive value (NPV). Logistic regression analysis findings are presented in Table 2. This analysis revealed that NLR values of 4.419 or higher had 4.28 times higher risk for appendicitis (*p* < 0.01), NPR values of 0.0327 or higher had 5.55 times higher risk for appendicitis (*p* < 0.01), NMR values of 8.29 or higher had 4.26 times higher risk for appendicitis (*p* < 0.01), PLR values of 130.47 or higher had 5.12 times higher risk for appendicitis (*p* < 0.01), and MLR values of 1.94 or lower had 7.7 times higher risk for appendicitis (p < 0.01). We used ROC (receiver operating characteristic) curves to graphically illustrate the relationship between the true‐positive rate (sensitivity) and the false‐positive rate (1‐specificity) for NLR, PLR, NMR, and NPR (Figure 2). The ROC curve for MLR is shown separately (Figure 3), and the corresponding AUC values are presented in Table 2.

**TABLE 1:** Inflammatory biomarkers.

	Appendicitis (*N* = 891)	NA (*N* = 136)	*p*
Neutrophils (K/μL) Mean (±SD) Median (95% CI)	11.85 (± 4.51) 11.70 (11.40–12.10)	7.41 (± 4.14) 6.70 (5.80–7.50)	< 0.001
Lymphocytes (K/μL) Mean (SD) Median (95% CI)	1.69 (±0.86) 1.50 (1.50–1.70)	2.29 (± 1.03) 2.20 (2.10–2.40)	< 0.001*
Monocytes (K/μL) Mean (SD) Median (95% CI)	0.98 (± 0.41) 0.90 (0.90–1.00)	0.83 (± 0.39) 0.80 (0.80–1.00)	0.001*
Platelets (K/μL) Mean (SD) Median (95% CI)	275.36 (± 72.73) 266 (260–271)	295.65 (± 90.17) 276.5 (262–303)	0.031*
CRP mg/L Mean (SD) Median (95% CI)	39.90 (± 64.88) 15.11 (13.27–17.37)	21.78 (± 31.48) 10.23 (4.88–12.11)	0.001*
Temp (Celsius) Mean (SD) Median (95% CI)	37.04 (± 0.53) 36.90 (36.90–37.00)	37.03 (± 0.59) 36.90 (36.90–37.10)	0.438*
NLR Mean (SD) Median (95% CI)	9.58 (±7.44) 7.56 (7.10–8.03)	4.45 (± 4.20) 2.85 (2.42–3.62)	< 0.001*
MLR Mean (SD) Median (95% CI)	2.02 (± 1.52) 1.67 (1.60–1.75)	3.09 (± 1.60) 2.79 (2.43–3.25)	< 0.001*
PLR Mean (SD) Median (95% CI)	207.09 (± 126.22) 174.62 (166.84–182.50)	156.73 (±97.87) 131.06 (117.37–141.33)	< 0.001*
NMR Mean (SD) Median (95% CI)	13.51 (± 8.25) 11.83 (11.44–12.21)	9.67 (± 5.41) 8.23 (7.43–9.83)	< 0.001*
NPR Mean (SD) Median (95% CI)	0.04 (± 0.02) 0.04 (0.04–0.05)	0.03 (± 0.01) 0.02 (0.02–0.03)	< 0.001

*Note:* A *p*‐value ≤ 0.05 was considered significant.

* The Mann–Whitney *U* Test for non‐parametric tests was performed.

**TABLE 2:** ROC analysis.

Biomarker	Cutoff	Area (AUC)	Asymptotic sig.	Sensitivity (%)	Specificity (%)	PPV (%)	NPV (%)	95% CI (Lower‐Upper)
*NLR*	≥ 4.42	0.772	< *0.001*	75.0	71.0	94.4	30.2	0.728–0.816
*MLR*	≤ 1.94	0.727	< *0.001*	74.3	59.5	92.3	26.1	0.680–0.768
*PLR*	≥ 130.47	0.645	< *0.001*	70.0	50.0	90.2	20.3	0.599–0.691
*NMR*	≥ 8.29	0.706	< *0.001*	82.3	50.7	91.6	30.4	0.655–0.758
*NPR*	≥ 0.0327	0.802	< *0.001*	75.2	77.2	95.6	32.2	0.761–0.843

## 4 Discussion

The accuracy of ultrasound in diagnosing appendicitis is highly dependent on the examiner's expertise, posing challenges in pediatric patients due to their smaller anatomical structures and limited cooperation. Given the increased risk of radiation‐induced malignancy in children, computed tomography (CT) is less favored despite its superior diagnostic clarity. In contrast, adults benefit from clearer anatomical visualization and more typical presentations, necessitating distinct diagnostic strategies for pediatric patients to minimize radiation exposure [5, 6].

Our findings demonstrate the moderate effectiveness of ultrasound in detecting appendicitis, with a sensitivity of 71.8% and a specificity of 44.8%. While ultrasound can identify a significant proportion of true cases, its limited specificity underscores the need for complementary diagnostic tools, such as blood biomarkers, to enhance diagnostic accuracy. Compared to previous studies, our results indicate better sensitivity but lower specificity, highlighting the variability in ultrasound performance across different clinical settings [7].

This study underscores the potential role of inflammatory markers in diagnosing appendicitis in pediatric patients. Elevated neutrophil‐to‐lymphocyte ratio (NLR), neutrophil‐to‐monocyte ratio (NMR), platelet‐to‐lymphocyte ratio (PLR), neutrophil‐to‐platelet ratio (NPR), and C‐reactive protein (CRP) were found to be significant indicators of appendicitis, reflecting the underlying inflammatory response triggered by bacterial infection. Although previous studies have demonstrated that the monocyte‐to‐lymphocyte ratio (MLR) tends to increase in infectious diseases such as pneumonia, tuberculosis, and sepsis, our findings reveal a statistically significant decrease in MLR among patients with appendicitis. This trend, also observed in another recent study, suggests that MLR behaves differently in appendicitis compared to other infections [16]. One possible explanation lies in the differing kinetics of immune cell responses: during acute bacterial infections, lymphocyte counts often decrease rapidly due to redistribution and apoptosis, whereas monocyte levels tend to rise more gradually, typically after the initial surge in neutrophils [16, 32]. This delayed monocyte response may result in a lower MLR during the early stages of inflammation, as seen in our appendicitis cohort. Further research focusing on the temporal evolution of MLR in acute infections could help clarify its potential role as a diagnostic marker [32, 33].

In our study we found that platelet counts were significantly different between groups (*p* = 0.031). A significant difference in PLR (*p* < 0.001) was observed, emphasizing the role of platelets in driving inflammation [34]. NLR emerged as one of the most reliable biomarkers, reflecting the balance between neutrophil‐driven inflammation and lymphocyte‐mediated immune regulation. NPR, which accounts for the relationship between neutrophils and platelets, also exhibited strong diagnostic potential, reinforcing the role of platelets as acute‐phase reactants in inflammation. CRP, a well‐established marker of systemic inflammation, further improved diagnostic accuracy when combined with other biomarkers [20, 21].

Interestingly, the absence of a significant temperature difference between appendicitis and non‐appendicitis groups suggests that early presentation or antipyretic use may obscure fever as a clinical sign. This finding highlights the limitations of relying on single parameters and supports the development of a multi‐marker scoring system to improve diagnostic precision. The identified cut‐off values for NLR (≥ 4.42), NPR (≥ 0.0327), NMR (≥ 8.29), PLR (≥ 130.47), and MLR (≤ 1.94) provide a balance between sensitivity and specificity. However, unlike the other biomarkers, MLR demonstrates increased sensitivity and specificity at lower levels. Integrating these biomarkers with clinical presentation and imaging findings may enhance diagnostic accuracy while reducing reliance on imaging, particularly in resource‐limited settings and in pediatric populations, where diagnosis is inherently more challenging than in adults.

Compared to established clinical scoring models such as the Alvarado score or the Appendicitis Inflammatory Response (AIR) score, our biomarker‐based model offers similar diagnostic metrics. For example, while the Alvarado score has an AUC of 0.790 and AIR has an AUC of 0.810 [18, 19], NPR and NLR in our analysis achieved AUCs of 0.802 and 0.772, respectively. However, unlike clinical scores which rely on subjective clinical parameters such as nausea or rebound tenderness, biomarkers provide objective, quantifiable metrics with high reproducibility.

Importantly, in this study, all surgical appendicitis cases, both simple and complicated, were included within the appendicitis group, reflecting real‐world emergency settings where such distinctions are not always evident preoperatively. This inclusive approach enhances generalizability but also underscores a limitation—biomarker performance in distinguishing between complicated and uncomplicated cases was not separately evaluated.

Other limitations include the retrospective single‐center design and the absence of external validation. Moreover, our analysis did not control for comorbidities or examine the influence of age stratification. Additionally, we did not evaluate time‐based changes in biomarker levels, which could influence diagnostic accuracy. Prospective multicenter trials comparing biomarker trends with imaging and clinical scores would provide more definitive insights.

## 5 Conclusion

This study highlights the diagnostic value of combining inflammatory markers—NLR, MLR, NMR, PLR, NPR, and CRP—in the preoperative evaluation of appendicitis in pediatric patients. By leveraging the complementary strengths of these biomarkers, a novel scoring system could enhance diagnostic accuracy, facilitate early intervention, and reduce reliance on imaging, particularly in resource‐limited settings. The unexpected inverse association observed with MLR underscores the need for further investigation into its role in the pathophysiology of appendicitis. Future multicenter, prospective studies are warranted to validate this scoring system, ensuring its clinical applicability across diverse healthcare environments.

## Author Contributions

Adir Alper and Osnat Zmora designed the study; Alper Adir and Lior Shlomov participated in data analysis; Alper Adir and Yizhak Aronov reviewed the manuscript. Yizhak Aronov and Lior Shlomov calculated the NLR, MLR, and PLR values. Adir Alper, Osnat Zmora, Lior Shlomov, and Yizhak Aronov participated in writing the draft of the manuscript. Adir Alper wrote the final article. Osnat Zmora was responsible for project administration and supervision. All authors contributed to the article and approved the submitted version.

## Ethics Statement

This study was approved by the Institutional Review Board (IRB) of Shamir Medical Center prior to its commencement. All procedures performed in this study were in accordance with the ethical standards of the institutional research committee and with the 1964 Helsinki Declaration and its later amendments.

## Consent

Informed consent was waived by the Institutional Review Board due to the retrospective nature of the study and the use of de‐identified patient data.

## Conflicts of Interest

The authors declare no conflicts of interest.

## Data Availability

The data that support the findings of this study are available from the corresponding author upon reasonable request. Due to ethical and privacy considerations, the data are not publicly accessible.
